# The oldest record of ornithuromorpha from the early cretaceous of China

**DOI:** 10.1038/ncomms7987

**Published:** 2015-05-05

**Authors:** Min Wang, Xiaoting Zheng, Jingmai K. O'Connor, Graeme T. Lloyd, Xiaoli Wang, Yan Wang, Xiaomei Zhang, Zhonghe Zhou

**Affiliations:** 1 Key Laboratory of Vertebrate Evolution and Human Origins of Chinese Academy of Sciences, Institute of Vertebrate Paleontology and Paleoanthropology, Chinese Academy of Sciences, Beijing 100044, China; 2 Institue of Geology and Paleontology, Linyi University, Linyi, Shandong 276000, China; 3 Tianyu Natural History Museum of Shandong, Pingyi, Shandong 273300, China; 4 Department of Biological Sciences, Faculty of Science, Macquarie University, Sydney, New South Wales 2019, Australia

## Abstract

Ornithuromorpha is the most inclusive clade containing extant birds but not the Mesozoic Enantiornithes. The early evolutionary history of this avian clade has been advanced with recent discoveries from Cretaceous deposits, indicating that Ornithuromorpha and Enantiornithes are the two major avian groups in Mesozoic. Here we report on a new ornithuromorph bird, *Archaeornithura meemannae* gen. et sp. nov., from the second oldest avian-bearing deposits (130.7 Ma) in the world. The new taxon is referable to the Hongshanornithidae and constitutes the oldest record of the Ornithuromorpha. However, *A. meemannae* shows few primitive features relative to younger hongshanornithids and is deeply nested within the Hongshanornithidae, suggesting that this clade is already well established. The new discovery extends the record of Ornithuromorpha by five to six million years, which in turn pushes back the divergence times of early avian lingeages into the Early Cretaceous.

## Abstract (web summary)

The origin and diversification of early birds remain unclear. Here, the authors report fossils from the oldest known ornithuromorph bird, recovered from the Huajiying Formation in China, which pushes the divergence of these and other early bird lineages back to the Jurassic–Cretaceous transition.

## Graphical abstract

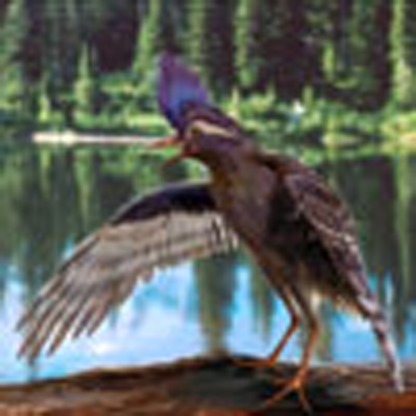

Ornithuromorpha is the clade of birds that all living birds but not Enantiornithes nest in refs 1, 2. Until now the earliest record of this group was from the lower Cretaceous Yixian Formation (125 Ma), the middle phase in the evolution of the Jehol Biota—the most important and diverse fossil avifauna known to science^1^,^3^,^4^. More than half the known diversity of Mesozoic birds belongs to this biota, which includes, from the oldest to the youngest, the *Protopteryx* horizon and the Yixian and Jiufotang Formations, which together encompass over ten million years of avian evolution (130.7–120 Ma)^5^. Bio- and lithostratigraphic works confirmed that the *Protoptery*x-bearing deposits in Sichakou basin of Fengning Country are referable to the interbedded shales from the lower part of the Huajiying Formation^5^,^6^. Recent ^40^Ar/^39^Ar dating produced a weighted mean age of 130.7 Ma for this horizon^7^. Therefore, the Huajiying Formation is the second oldest avian-bearing deposit in the world, only after the Upper Jurassic Solnhofen Limestones that preserve *Archaeopteryx* in Germany^6^. Only three avian taxa have been collected from this horizon: the basal confuciusornithiform *Eoconfuciusornis* and two enantiornithines *Protopteryx* and *Eopengornis*^8^,^9^,^10^. Here we report on the first two ornithuromorph fossils from the same locality as the holotype of *Protopteryx fengningensis* in Sichakou basin^8^, which constitute the earliest known record of Ornithuromorpha. These two specimens are referable to a single taxon, which appears to be deeply nested within the Hongshanornithidae, a fairly diverse clade of small, specialized wading ornithuromorphs. Currently, four hongshanornithids, *Hongshanornis longicresta*, *Longicrusavis houi*, *Parahongshanornis chaoyangensis* and *Tianyuornis cheni*, have been reported from the Yixian and Jiufotang Formations in Inner Mongolia and Liaoning^2^,^11^,^12^,^13^,^14^. These new specimens reported here push back the first appearance datum of Ornithuromorpha by approximately six million years.

## Results

### Systematic paleontology

         Aves Ornithothoraces Ornithuromorpha Hongshanornithidae

 **Type genus.** *Hongshanornis*, Zhou and Zhang^11^.

*Archaeornithura meemannae* gen. et sp. nov.

 **Etymology.** The generic name is derived from Greek ‘*Archae*' and ‘*ornithura*', meaning ‘ancient ornithuromorph'. The specific name is in honour of Dr Meemann Chang for her continuous support of the study of the Jehol Biota.

**Figure 1:**
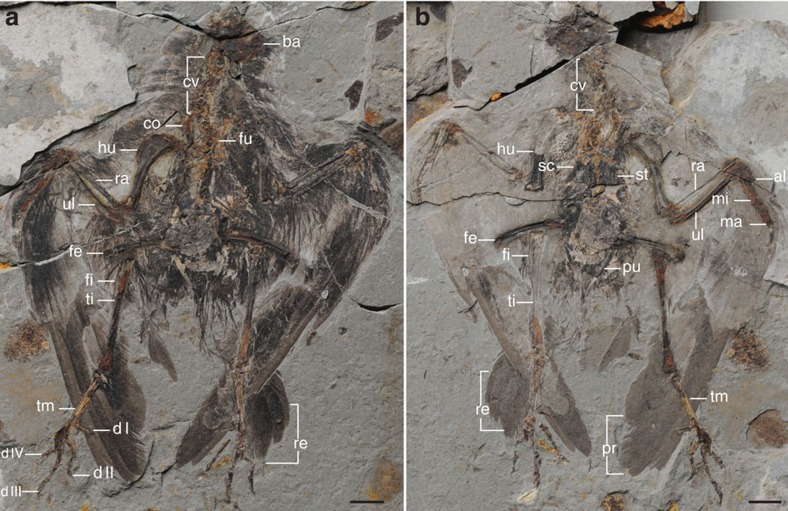
Holotype of *Archaeornithura meemannae* gen. et sp. nov., STM7-145. (**a**) Main slab; (**b**) counter slab. Anatomical abbreviations: al, alular digit; ba, basicranium; co, coracoid; cv, cervical vertebrae; d I–IV, pedal digit I–IV; fe, femur; fi, fibula; fu, furcula; hu, humerus; ma, major digit; mi, minor digit; pr, primary remiges; pu, pubis; ra, radius; re, rectrices; sc, scapula; st, sternum; ti, tibiotarsus; tm, tarsometatarsus; ul, ulna. Scale bars, 10 mm.

 **Holotype.** An articulated partial skeleton with feathers (STM7-145), housed at the Tianyu Natural History Museum of Shandong (STM), China (Fig. 1).

 **Paratype.** An articulated partial skeleton with feathers (STM7-163; Supplementary Fig. 1).

 **Locality and horizon.** *Protopteryx* horizon in Sichakou basin, Fengning County, Hebei, northeastern China; Lower Cretaceous Huajiying Formation^6^,^7^.

 **Differential diagnosis.** The new taxon is referable to the Hongshanornithidae and can be distinguished from the known hongshanornithids by the following combined features: it differs from *Hongshanornis* and *Longicrusavis*, in that the cranial margin of the sternum is strongly vaulted; it is distinguished from *Hongshanornis* and *Parahongshanornis*, in that the zyphoid process of the sternum is well developed and squared; it differs from other hongshanornithids except *Longicrusavis*, in that the alular digit extends further distally than the major metacarpal; it is distinguishable from other hongshanornithids except *Parahongshanornis*, in that the penultimate phalanx of the major digit is longer than the preceding phalanx; and the new taxon has proportionally shorter femur relative to the tarsometatarsus.

### Description

**Figure 2:**
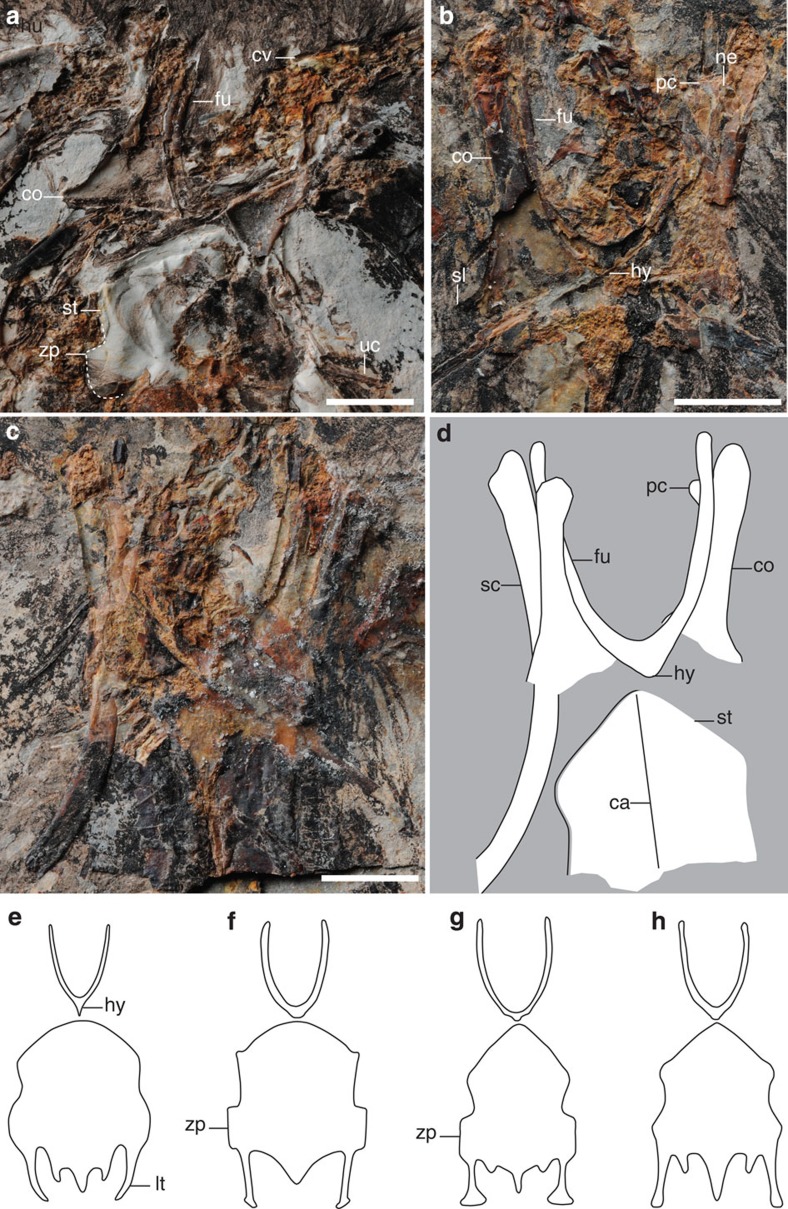
Pectoral girdle and sternum of *Archaeornithura meemannae* gen. et sp. nov., in comparison with other hongshanornithids. (**a**) STM7-163, counter slab (zyphoid process of the sternum is outlined by a dash line); (**b**) STM7-145, main slab; (**c**) photograph and (**d**) line drawing of STM7-145, counter slab. Line drawing (not scaled) of furcula and sternum of other hongshanornithids: (**e**) *Hongshanornis longicresta*; (**f**) *Longicrusavis houi*; (**g**) *Tianyuornis cheni*; (**h**) *Parahongshanornis chaoyangensis*. Anatomical abbreviations: ca, carina; co, coracoid; cv, cervical vertebrae; fu, furcula; hy, hypocleidium; lt, lateral trabecula of the sternum; ne, supracoracoidal nerve foramen; pc, procoracoid process; sc, scapula; sl, sternocoracoidal process; st, sternum; uc, uncinate process; zp, zyphoid process of the sternum. Scale bars, 5 mm.

**Figure 3:**
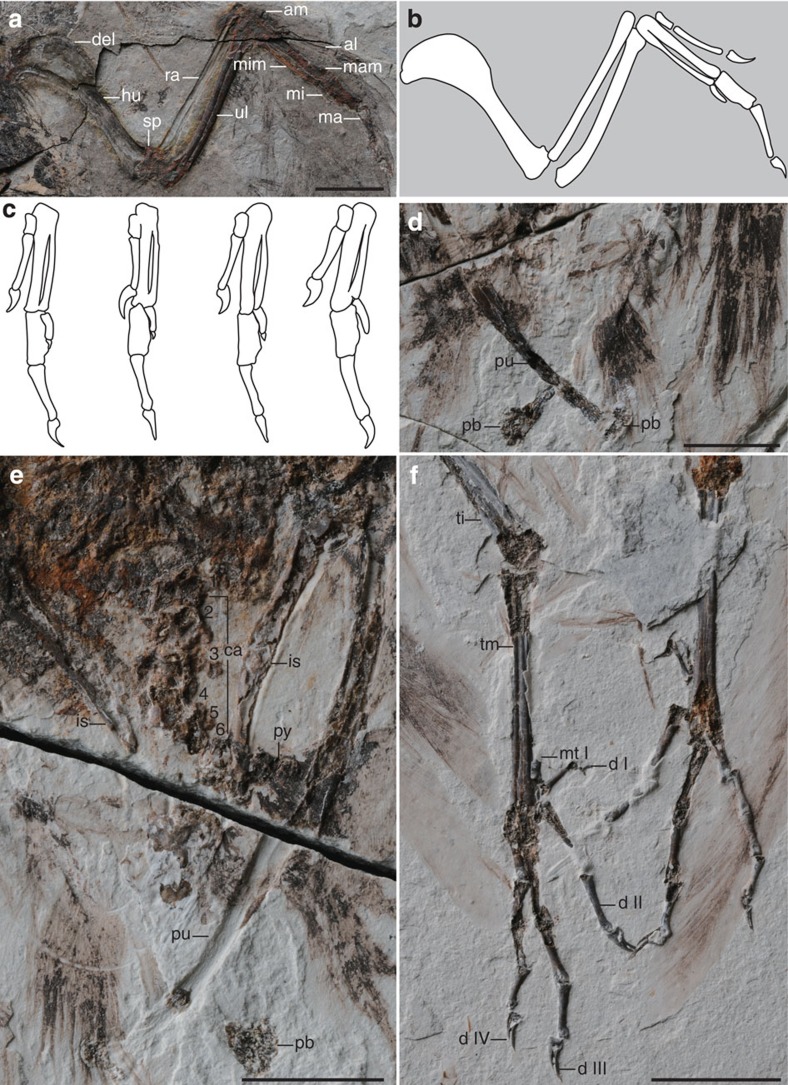
Detail anatomy of *Archaeornithura meemannae* gen. et sp. nov. (**a**) Photograph and (**b**) line drawing of the left wing, STM7-145, counter slab; (**c**) line drawing of hands of other hongshanornithids (not scaled; from left): *Hongshanornis longicresta*, *Longicrusavis houi*, *Tianyuornis cheni*, *Parahongshanornis chaoyangensis*; (**d**) STM7-163, counter slab; (**e**) STM7-163, main slab; (**f**) feet, STM7-163, main slab. Anatomical abbreviations: al, alular digit; am, alular metacarpal; ca, caudal vertebrae (six vertebrae counted); del, deltopectoral crest; d I–IV, pedal digit I–IV; hu, humerus; is, ischium; ma, major digit; mam, major metacarpal; mi, minor digit; mim, minor metacarpal; mt I, metatarsal I; pb, pubic boot; pu, pubis; py, pygostyle; ra, radius; sp, supracondylar process; ti, tibiotarsus; tm, tarsometatarsus; ul, ulna. Scale bars, 10 mm (**a**,**f**), 5 mm (**d**,**e**).

The skull is not well preserved in either specimen (Fig. 1; Supplementary Fig. 1). The cervical vertebrae are poorly preserved in articulation with the skull; 10 vertebrae are preserved including the ring-like axis, although the exact position of the cervicothoracic transition cannot be determined due to poor preservation. The vertebrae are clearly not elongated, being approximately equal in width and length, as in other hongshanornithids (Figs 1 and 2b,c; Supplementary Fig. 1). The thoracic vertebrae are only preserved in the counter slab of STM7-145, covered by the sternum. The synsacrum appears to comprise 9–10 vertebrae, although preservation makes this estimate equivocal. Six free caudals are followed by a poorly preserved pygostyle (Fig. 3e). The transverse processes of the caudal vertebrae are less than the width of the centrum in length and caudolaterally directed, at least in the first free caudal. Two well-preserved uncinate processes are preserved in the counter slab of STM7-163, and they are straight and tapered (Fig. 2a). Although not in articulation with the ribs, we infer they would have crossed at least one rib, reaching and potentially crossing a second. Four sets of gastralia are preserved in articulation in STM7-163 (Supplementary Fig. 2b).

The scapula is curved, as in other ornithuromorph birds, but the distal end is not well preserved in either specimen (Figs 1 and 2c). The furcula is typically hongshanornithid: delicate, U-shaped, with tapered omal margins and a small tubercle-like hypocleidium as in *Tianyuornis* and *Parahongshanornis*^12^,^13^,^14^, which, however, is short and sharply tapered in *Hongshanornis* (Fig. 2)^11^. In contrast, a hypocleidium is absent from most other Cretaceous ornithuromorphs, although a short one has been reported in *Schizooura*^15^. The furcular symphysis is much thicker craniocaudally compared with the omal rami as in *Hongshanornis*, whereas the furcula appears to be nearly of equal thickness throughout in *Longirostravis* and *Tianyuornis*^12^,^13^. The coracoid is narrow along the proximal half, and wide distally with a well-developed sternocoracoidal process (Fig. 2b), as in hongshanornithids and most other ornithuromorphs^2^,^12^,^13^,^14^. Although poorly preserved, a procoracoid process is visible on the right coracoid in the counter slab of both specimens; it is medially oriented although the distal half appears more cranially directed (Fig. 2b). This process is perforated at the base by a small circular supracoracoidal nerve foramen. The coracoid is preserved in the dorsal view in the counter slab of STM7-163 (Fig. 2b); the acrocoracoid is blunt, the scapular cotyla is deeply concave, and the laterally positioned glenoid is developed as a weak convexity. Distally, the corpus is concave, more so than the condition in *Yixianornis* but not so deeply as in some Late Cretaceous enantiornithines^16^. The sternum is not well preserved, but a fragment in STM7-163 indicates the rostral margin is vaulted as in *Tianyuornis* and *Parahongshanornis* (Fig. 2d,g,h)^13^,^14^, but more pointed than in *Longicrusavis* and *Hongshanornis*, in which the cranial margin is broad and parabolic (Fig. 2e,f)^2^,^11^,^12^. A well-developed zyphoid process is present and squared in shape, as in *Longicrusavis* and *Tianyuornis* (Fig. 2a,f,g)^12^,^13^, whereas the process is absent in *Hongshanornis* and *Parahongshanornis*, and the corresponding lateral margin only bulges laterally (Fig. 2e,h)^11^,^14^. A second fragment in the same slab indicates that the lateral trabecula was short and narrow as in other hongshanornithids, with a distinct triangular distal expansion similar to that of *Tianyuornis* but smaller (Supplementary Fig. 2a)^13^.

The humerus is short and robust with a large rounded deltopectoral crest that extends 44% the length of the humerus and slightly exceeds the width of the shaft, as in other hongshanornithids (Fig. 3a). The distal margin is perpendicular to the shaft. The condyles are well developed and bulbous, and a dorsal supracondylar process is present as in other hongshanornithids (Fig. 3a; Supplementary Fig. 2d)^2^,^12^; however, the process has so far been reported only in *Ichthyornis* among other Mesozoic birds^2^,^17^. The ulna is bowed and more robust than the straight radius as in all basal birds. An olecranon process is not developed. The carpometacarpus appears to be fused proximally but not distally in STM7-145. All the metacarpals are straight, and the minor metacarpal is less than half the thickness of the major metacarpal. The alular metacarpal bears a small extensor process that is less than half the width of the articular facet of this metacarpal. The alular digit is long, with half of the ungual extending just beyond the distal margin of the major metacarpal as in *Longicrusavis*, whereas in other hongshanornithids, only the tip of the ungual slightly surpasses the distal end of the major metacarpal (Fig. 3a,c). The ungual phalanx of the alular digit is larger and more recurved than that of the major digit, as in other hongshanornithids^2^,^11^,^12^,^13^,^14^. The first phalanx of the major digit is mediolaterally compressed and caudally expanded, as in other ornithuromorphs. As in *Parahongshanornis*, the penultimate phalanx is longer than the preceding phalanx, whereas these two phalanges are subequal in length in other hongshanornithids (Table 1). The minor digit only preserves a single reduced wedge-shaped phalanx that tapers distally. A second phalanx, which is extremely reduced in other hongshanornithids^2^,^11^,^12^,^13^,^14^, may have being missing due to preservation. Similar to other hongshanornithids, the forelimb is much shorter than the hindlimb, with an intermembral index (humerus+ulna/femur+tibiotarsus) of ∼0.84 (Table 1), whereas the forelimb is typically longer in other Early Cretaceous ornithuromorphs^18^,^19^.

The pelvic girdle is not well preserved in either specimen; the ilium is displaced cranially in STM7-145. Visible in the main slab, the cranial margin is convex, while the lateral margin proximal to the acetabulum is concave; the two margins are separated by a weakly developed ventral hook present in most ornithuromorphs and some enantiornithines. The pubes are long and contact along their distal tenth but remain unfused, as in *Hongshanornis* (Supplementary Fig. 2c). Although incomplete, the distal ends of pubes are well preserved in lateral and medial views in the counter slab of STM7-163, revealing a small distally expanded boot as in *Hongshanornis* and *Parahongshanornis* (unclear in *Tianyuornis* and *Longirostravis*; Fig. 3d,e)^2^,^14^. The ischia appear to be just over half the length of the pubes, straight, narrow and tapered along their distal halves (Fig. 3e), lacking the low dorsal process located mid-corpus and the concave ventral margin present in most other ornithuromorphs, including *Yixianornis* and *Piscivoravis*^20^,^21^.

The femora are short and fairly robust, approximately equal in length to the tarsometatarsi; in contrast, the femur is considerably longer than the latter in most other basal birds, including *Jeholornis*, *Sapeornis*, *Confuciusornis*, enantiornithines and ornithuromorphs^18^,^19^. The tibiotarsus is proportionally shorter than other hongshanornithids relative to the tarsometatarsus. As in *Longicrusavis*, the fibula is preserved bowing out from the tibiotarsus, not appressed against it, and only appears to extend to the midshaft of the tibiotarsus^12^. The proximal medial surface bears a shallow excavation, visible on the left side in the counter slab of STM7-163.

The tarsometatarsi are well preserved in the main slab of STM7-163 (Fig. 3f). They are fully fused, although the individual metatarsals can be distinguished. Overall, the foot is very similar to other hongshanornithids^2^,^11^,^12^,^13^,^14^. The proximal half of metatarsal III is plantarly displaced relative to metatarsals II and IV, as in all ornithuromorphs. Metatarsal III is the longest and metatarsals II and IV end approximately at the same level. The hallux is small and placed above the trochlea of the other digits as in most ornithuromorphs including other hongshanornithids. The first phalanx is approximately as long as metatarsal I itself; the ungual phalanx is more strongly recurved than that of the other digits. As in other hongshanornithids, the phalanges decease in length distally, digit III is the longest and digit II is substantially shorter than IV.

**Figure 4:**
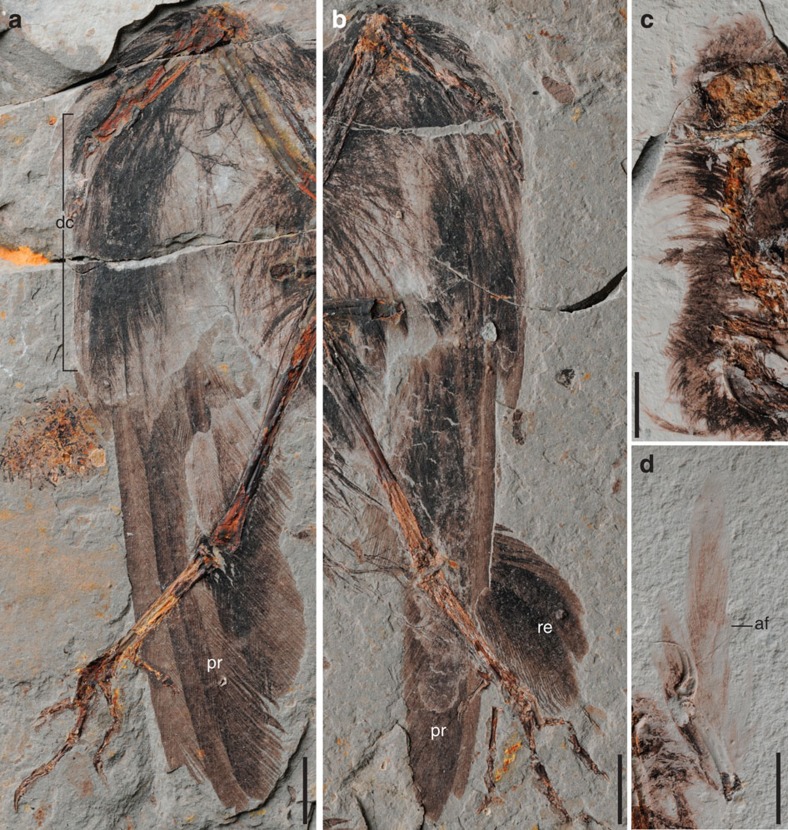
Plumage of *Archaeornithura meemannae* gen. et sp. nov. (**a**) Left wing, STM-7-145, main slab; (**b**) right wing, STM-7-145, main slab; (**c**) covert feathers over the skull and neck, STM 7-163, counter slab; (**d**) alular feathers on the left alular digit, STM7-163, main slab. Abbreviations: af, alular feather; dc, dorsal coverts; pr, primary remiges; re, rectrices. Scale bars, 10 mm (**a**–**c**), 5 mm (**d**).

Both specimens preserve nearly complete plumage (Figs 1 and 4; Supplementary Fig. 1). Six long asymmetrical primary remiges are preserved in the left wing of the main slab in the holotype (Fig. 4a); the second and third feathers are the longest. The primary remiges are overlain by a layer of short feathers, which measure just under half the length of the primary remiges themselves—we interpret these feathers as the dorsal coverts (Fig. 4a,b). They appear to be symmetrical, definitely lacking the strong asymmetry present in the primary remiges in which the leading edge vane is less than one-third the width of the trailing edge vane (Fig. 4a). Portions of a few secondary feathers are also preserved; these appear to be narrower than the primary remiges and symmetrical with rounded distal margins. An alula is preserved in both specimens, composed of at least three feathers in STM7-163, visible where the alular digit is disarticulated on the left side (Fig. 4d). The rounded distal margins of three large symmetrical pennaceous feathers are preserved near the right foot in STM7-145 (Figs 1 and 4b). These feathers are staggered so that each medial feather ends distal to the lateral feather, suggesting that these feathers represent the distal portion of an incomplete fan-shaped array of rectrices, like that present in *Hongshanornis* (IVPP V14533 and DNHM D2945/6)^2^.

Short rachis-less covert feathers are found all over the body, particularly well preserved in STM7-163 (Supplementary Fig. 1). These feathers cover the head, neck, shoulders, extend off the proximal ulna and humerus and line the caudal end of the body (Fig. 4c; Supplementary Fig. 1). These feathers are notably absent from the distal three-quarters of the tibiotarsus in both specimens (Figs 1 and 4a; Supplementary Fig. 1), consistent with the wading habitat inferred for hongshanornithids^12^.

## Discussion

**Figure 5:**
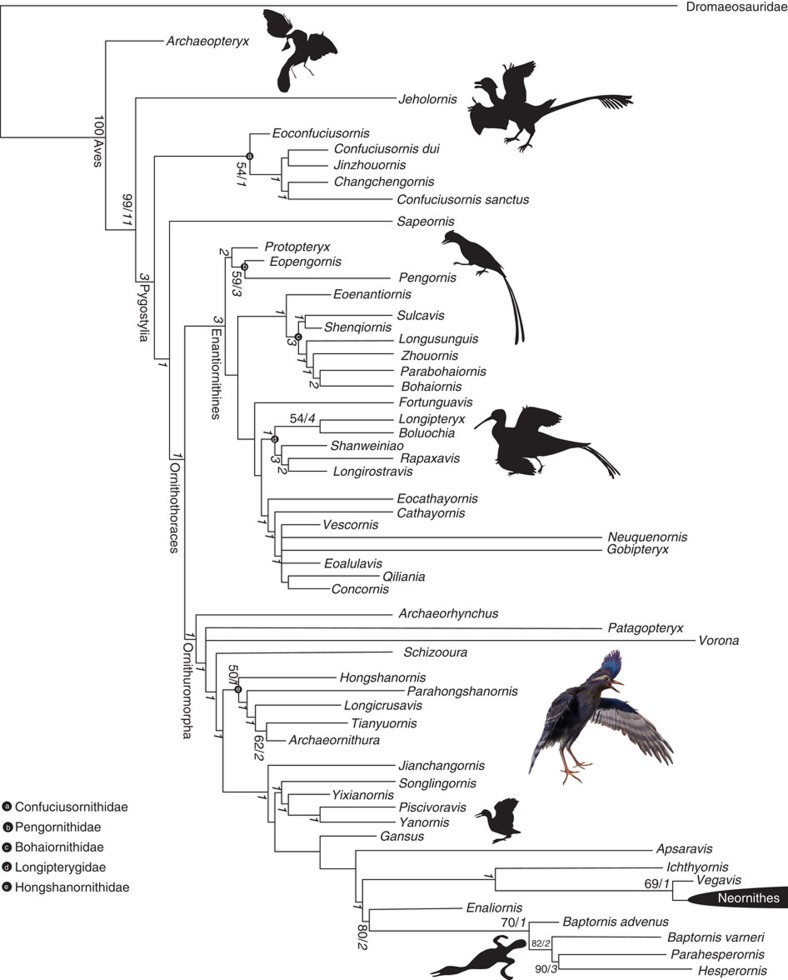
Cladogram showing the systematic position of *Archaeornithura meemannae* among Mesozoic birds. Bootstrap and bremer values are labelled to the corresponding nodes in normal and bold italic formats, respectively.

*Archaeornithura* is referable to the Hongshanornithidae, which is distinguishable from other Cretaceous ornithuromorphs by the following synapomorphies: the U-shaped furcula bears a tubercle-like hypocleidium; the humerus has a well-developed supracondylar process; the manus is longer than the humerus; the forelimb is shorter than the hindlimb with an intermembral index of about 0.8; and the femur and tarsometatarsus are subequal in length, while the former is considerably longer in most other basal birds. Phylogenetic analysis was conducted using a comprehensive matrix targeted at Mesozoic birds (58 taxa and 262 characters; Supplementary Data 1 and Supplementary Data 2)^22^; the results confirm the close affinity between the new specimens and younger hongshanornithids, resolving all purported members together in a clade, the Hongshanornithidae (Fig. 5; Supplementary Fig. 3). This represents the most diverse recognized clade of Early Cretaceous ornithuromorphs. Synapomorphies of the Hongshanornithidae retained from the analysis include: teeth present throughout the premaxillary (character 4:0); mandibular ramus sigmoidal in shape (character 42:1); acromion process of scapula nearly parallel to the scapular shaft in costal or lateral view (character 98:1); dorsal supracondylar process of humerus developed (character 134:2); second phalanx of the major digit longer than the proximal one (character 171:0); intermembral index between 0.7 and 0.9 (character 177:1); the claw of the fourth pedal digit smaller than that of other digits (character 256:1); and the ratio (length of tibiotarsus/tarsometatarsus) between 2 and 1.6 (character 257:1). *Archaeornithura* is resolved to be the sister taxon to the younger *Tianyuornis* and this clade is supported by two synapomorphies: outermost trabecula of sternum with a simple bulb-like distal expansion (character 113:1; Supplementary Fig. 2a) and metatarsals II–IV partially fused with discernible sutural contacts (222:1; Fig. 3f). It is notable that, despite being the oldest recognized ornithuromorph, *Archaeornithura* is deeply nested within the Hongshanornithidae, which itself is more deeply nested within Ornithuromorpha than some taxa known entirely from younger deposits^15^,^17^,^18^,^21^,^22^. These inconsistencies between stratigraphy and phylogeny require the presence of ghost lineages and a much earlier origination date for the Ornithuromorpha, which in turn pushes back the divergence time with enantiornithines and other primitive avian lineages.

*Archaeornithura* preserves fairly advanced plumage including a well-developed alula and fan-shaped rectrices (Figs 1 and 4; Supplementary Fig. 1). Both the alula (bastard wing) and a fan-shaped tail are aerodynamically important for living birds during slow flight and increases manoeuvrability^23^,^24^. These advanced feather structures are inferred to be plesiomorphic at least to Ornithothoraces^2^,^10^; an alula is also present in the basal enantiornithine *Protopteryx* from the same locality^8^. Because the alular digit is well developed in both hongshanornithids and *Protopteryx* (extends beyond the distal end of the major metacarpal), the morphology of the alula would be expected to somewhat differ from that in living birds. However, preservation of the alula in a Mesozoic bird has never been clearer than as preserved in STM7-163 (Fig. 4d), which reveals a morphology that is remarkably similar to that in living birds: it is formed by at least three feathers (three to five in living birds), with the short proximal feather ending level with the alular digit and the longer feathers extending to the end of the major digit. Both *Archaeornithura* and *Hongshanornis* preserve a fan-shaped array of rectrices^2^, a feature plesiomorphic to Ornithuromorpha and apparently lost in Enantiornithes^10^. Enantiornithines and the Confuciusornithiformes typically have only a pair of elongated rachis-dominant rectrices^8^,^9^,^25^,^26^. The rachis of these feathers is proportionately wide and always preserves a longitudinal stripe, which is interpreted as a central groove in some studies^10^,^26^. More recently, the same striped morphology was recognized in the primary feathers of *Confuciusornis* and enantiornithines, supporting hypotheses that rachis-dominated feathers are modified flight feathers^10^. *Archaeornithura* documents this feather morphology for the first time in the Ornithuromorpha; a medial stripe is visible in the rachises of some of the primary remiges. Although evidence from *Archaeopteryx* indicates modern feather morphology including fully pennaceous coverts and rachises that clearly lacked a longitudinal groove^27^, these features are absent in Jehol birds despite the huge wealth of specimens^25^,^26^. This suggests that modern feather morphologies evolved independently within the *Archaeopteryx* lineage and a derived subset of ornithuromorphs.

Bird fossils are extremely rare in the Mesozoic fossil record, and until the wealth of specimens discovered from Early Cretaceous deposits in northeastern China, very little was known about the early evolution of birds^1^,^3^,^4^. The Jehol Biota encapsulates a unique window into the biology and morphology of the oldest known avifauna; however, this fauna is clearly already well within the diversification of birds, given that both ornithothoracine clades are present^3^,^28^. Until now no ornithuromorphs had been described from the Huajiying Formation, which preserves very few fossil birds^10^; because diversity is low and geographic area is restricted, the Huajiying Formation is interpreted as the earliest stage in the diversification of the Jehol Biota. However, the discovery of a new species belonging to the specialized clade of waders—the Hongshanornithidae, indicates that ornithuromorphs themselves were already quite specialized at this point in their evolution. This also strongly supports inferences that this clade originated in a semi-aquatic environment^1^,^29^,^30^.

## Methods

### Provenance of the two specimens of *A. meemannae*

The two specimens (STM7-145, 7-163) of *A. meemannae* were acquired by the Tianyu Natural History Museum of Shandong from a fossil dealer. The dealer confirmed that the fossils were collected from the same locality of the holotype of *Protopteryx fengningensis* (IVPP V11665) in Sichakou basin of Fengning Country, Hebei Province, northeastern China. Lithostratigraphic and biostratigraphic fieldworks have been performed in the basin for years, and confirm that the *Protopteryx*-bearing horizon belongs to the Lower member of the Huajiying Formation^5^,^6^,^8^. The Cretaceous Strata cropping out in Sichakou basin consist of the Huajiying Formation and the overlying Qingshila Formation; the Huajiying Formation consists of lacustrine deposits with abundant pyroclastics, and all the known fossil birds, including the two specimens of *A. meemannae*, were unearthed from the lower part of the Huajiying Formation (‘Sichakou sedimentary member' in ref. 6). Currently, no fossil birds have been reported from the Qingshila Formation. A weighted mean age of 130.7 Ma was reported using ^40^Ar/^39^Ar dating of the K-feldspars samples from the interbedded tuffs about 6 m below the *Protopteryx*-bearing layer^7^, largely consistent with previous results using the SHRIMP U-Pb method^31^. The two specimens of *A. meemannae* are preserved in slabs that are identical to the matrixes containing the holotype of *Protopteryx fengningensis*, *Eoconfuciusornis zhengi* and *Eopengornis martini*, in that all the slabs are composed of tufaceous siltstones, which are grey in colour and rigid in nature, consistent with mineralogical composition of the birds-bearing deposits from the lower part of the Huajiying Formation^6^. We have compared hundreds fossil birds, housed in the Institute of Vertebrate Paleontology and Paleoanthropology, which are unearthed from the Yixian and Jiufotang Formations from a wide geological area in Jehol Biota. We have noticed that these slabs are off-white (rather than grey as in the *Archaeornithura*-bearing slabs) in colour and mainly composed of weakly laminated siliciclastic sandstones and shales, which are conspicuously softer than the bird-bearing slabs from the Huajiying Formation (including the slabs containing *A. meemannae*). In addition, we have requested the dealer to show us the exact locality where these two specimens were unearthed, and confirmed that they were collected from the Huajiying Formation in Sichakou basin. Detail comparisons and geological correlation indicate that the slabs preserving the two specimens of *A. meemannae* are identical in all visible lithological features to the sediments of the lower part of the Huajiying Formation. Therefore, the provenance of the two specimens of *A. meemannae* can be justified. The two specimens of *A. meemannae* are housed in the Tianyu Natural History Museum of Shandong and are publicly accessible.

### Phylogenetic analysis

Phylogenetic analysis was performed using the modified data set of Wang *et al*.^22^, a comprehensive morphological character matrix targeting the phylogeny of Mesozoic birds (Supplementary Data 1 and 2. *Rahonavis* was removed from the data set, given the fact that recent studies indicate that *Rahonavis* is an unenlagiine dromaeosaurid rather than a bird^32^,^33^. Two enantiornithines, *Elsornis* and *Iberomesornis*, were removed because of their incompleteness. Two recently reported Jehol birds, *Eopengornis martini* (enantiornithine) and *Piscivoravis lii* (ornithuromorph), were added and scored from their holotypes (STM24-1, IVPP V17078)^10^,^21^. All the known hongshanornithids were included, and *Archaeornithura*, *Parahongshanornis* and *Tianyuornis* were coded from their corresponding holotypes. The revised data matrix consists of 58 taxa (55 are Mesozoic birds), scored across 262 morphological characters. Phylogenetic analysis was conducted using PAUP software package version 4.0b10 (ref. 34), with the following settings: all characters equally weighted; unconstrained heuristic search starting with Wagner trees; 1,000 replicates of random stepwise addition (branch swapping: tree-bisection-reconnection) holding 10 trees at each step; and branches are collapsed to create polytomies if the minimum branch length is equal to zero. Bootstrap and Bremer values were calculated as indices of support. Bootstrap values were performed using the TNT software package (ref. 35) with default setting, except that 1,000 replicates were used. Only nodes with bootstrap values greater than 50% are shown in Fig. 5. Bremer values were calculated using the bremer scripts embedded in TNT.

The phylogenetic analysis produced four most parsimonious trees of 997 steps, and had a consistency index of 0.367 and retention index of 0.684 (Supplementary Fig. 3). The strict consensus tree is well resolved, and the new cladogram is essentially consistent with previous studies with regards to the placement of major clades^10^,^17^,^18^,^22^,^29^,^36^. In the strict consensus tree, *Longicrusavis*, *Parahongshanornis* and *Hongshanornis* form the successive outgroups to *Tianyuornis*+*Archaeornithura* clade, and these five taxa together constitute a clade, the Hongshanornithidae (Fig. 5).

### Nomenclatural Acts

This published work and the nomenclatural acts it contains have been registered in ZooBank, the proposed online registration system for the International Code of Zoological Nomenclature. The ZooBank LSIDs (Life Science Identifiers) can be resolved and the associated information viewed through any standard web browser by appending the LSID to the prefix ‘ http://zoobank.org/'. The LSIDs for this publication are: urn:lsid:zoobank.org:pub:B55C95A7-1AB4-4645-BEA4-FC7EE139C4A7.

## Additional information

**How to cite this article:** Wang, M. *et al*. The oldest record of ornithuromorpha from the early cretaceous of China. *Nat. Commun*. 6:6987 doi: 10.1038/ncomms7987 (2015).

## Supplementary Material

Supplementary Figures Supplementary Figures 1-3

Supplementary Data 1 Description of morphological character used in the phylogenetic analysis

Supplementary Data 2 Morphological character scorning in the phylogenetic analysis

## Figures and Tables

**Table 1:** Comparative measurements of *Archaeornithura meemannae* gen. et sp. nov. and other hongshanornithid taxa.

Elements	STM 7-145	STM 7-163	IVPP V 14533	DNHM D2945	PKUP 1069	PMOL-AB00161	STM7-53
Coracoid	15.4	12.7		11.7	12.7	14.7	
Scapula	>23.2			22.4	23.1	>24.5	
Humerus	25.9	27.5	26.0	22.9	26.0	29.4	25.4
Ulna	25.8	28.3	24.4	23.9	25.0	28.4	26.4
Radius	23.9	26.0	23.0	23.1	24.0	26.8	24.6
Carpometacarpus	13.1	13.8	11.8	12.6	13.1	11.4	13.2
Alular digit 1	6.0	7.6	6.3	6.6	6.9	6.7	6.6
Alular digit 2	3.0	3.9	2.8	2.8	4.3	2.7	2.9
Major digit 1	6.6	6.8	5.8	6.6	7.0	7.1	6.9
Major digit 2	7.6	7.0	5.9	6.8	7.3	8.0	7.0
Major digit 3	2.4	3.0	2.5		3.4	2.6	
Minor digit 1	3.3		2.8	4.0	3.2,	3.5	3.3
Femur	23.8		22		24.3	24.8	24.8
Tibiotarsus	38.0	37.5	38	34.6	37.6	41.3	39.0
Tarsometatarsus	23.0		21.0	19.1	21.5	21.2	23.1
(Humerus+ulna)/(femur+tibiotarsus)	0.84		0.84		0.82	0.87	0.81

*Archaeornithura meemannae* (STM7-145, STM7-163), *Hongshanornis longicresta* (holotype, IVPP V 14533; paratype, DNHM D2945), *Longicrusavis houi* (holotype, PKUP V1069), *Tianyuornis cheni* (holotype, STM7-53) and *Parahongshanornis chaoyangensis* (holotype, PMOL-AB00161). Lengths are measured in millimetres.
