# Supplementary material for: The oldest record of ornithuromorpha from the early cretaceous of China
Source: Nat Commun. 2015 May 5;6:6987. doi: 10.1038/ncomms7987 (PMC5426517; doi:10.1038/ncomms7987)
Supplement: Supplementary Figures — 1-3 [file ncomms7987-s1.pdf]

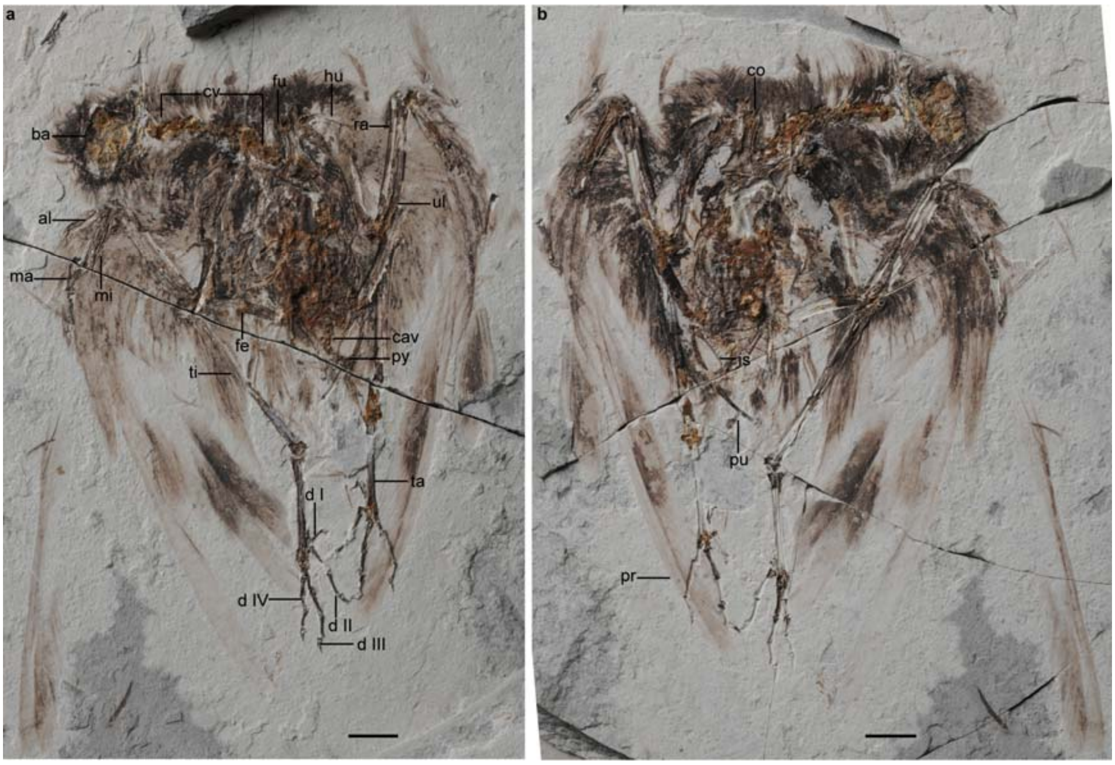

Supplementary Figure 1. Paratype of *Archaeornithura meemannae* gen. et sp. nov. (**a**) STM7-163, main slab; (**b**) counter slab. Anatomical abbreviations: al, alular digit; ba, basicranium; cav, caudal vertebrae; co, coracoid; cv, cervical vertebrae; d I–IV, pedal digit I–IV; fe, femur; fu, furcula; ga, gastralia; hu, humerus; is, ischium; ma, major digit; mi, minor digit; pr, primary remiges; pu, pubis; py, pygostyle; ra, radius; sc, scapula; ta, tarsometatarsus; ti, tibiotarsus; ul, ulna. Scale bars, 10 mm.

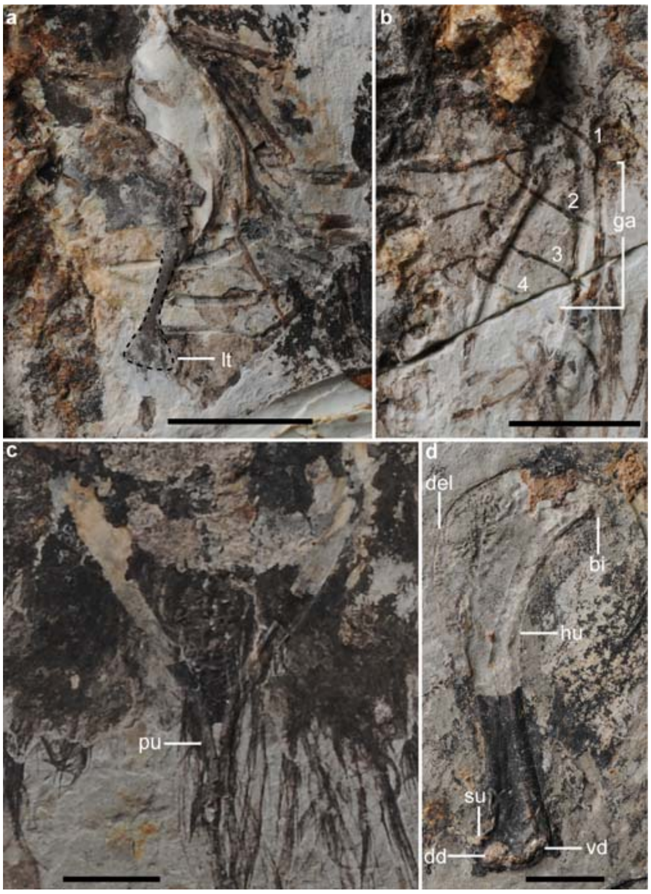

Supplementary Figure 2. Detail anatomy of *Archaeornithura meemannae* gen. et sp. nov. (**a**) Lateral trabecula of the sternum (outlined by dash line), STM7-163, counter slab; (**b**) four sets of gastralia, STM7-163, counter slab; (**c**) pubes, STM7-145, main slab; (**d**) right humerus in cranial view, STM7-145, counter slab. Anatomical abbreviations: bi, bicipital crest; dd, dorsal condyle; del, deltopectoral crest; ga, gastralia; hu, humerus; lt, lateral trabecula of the sternum; pu, pubis; su, dorsal supracondylar process; vd, ventral condyle. Scale bars, 5 mm.

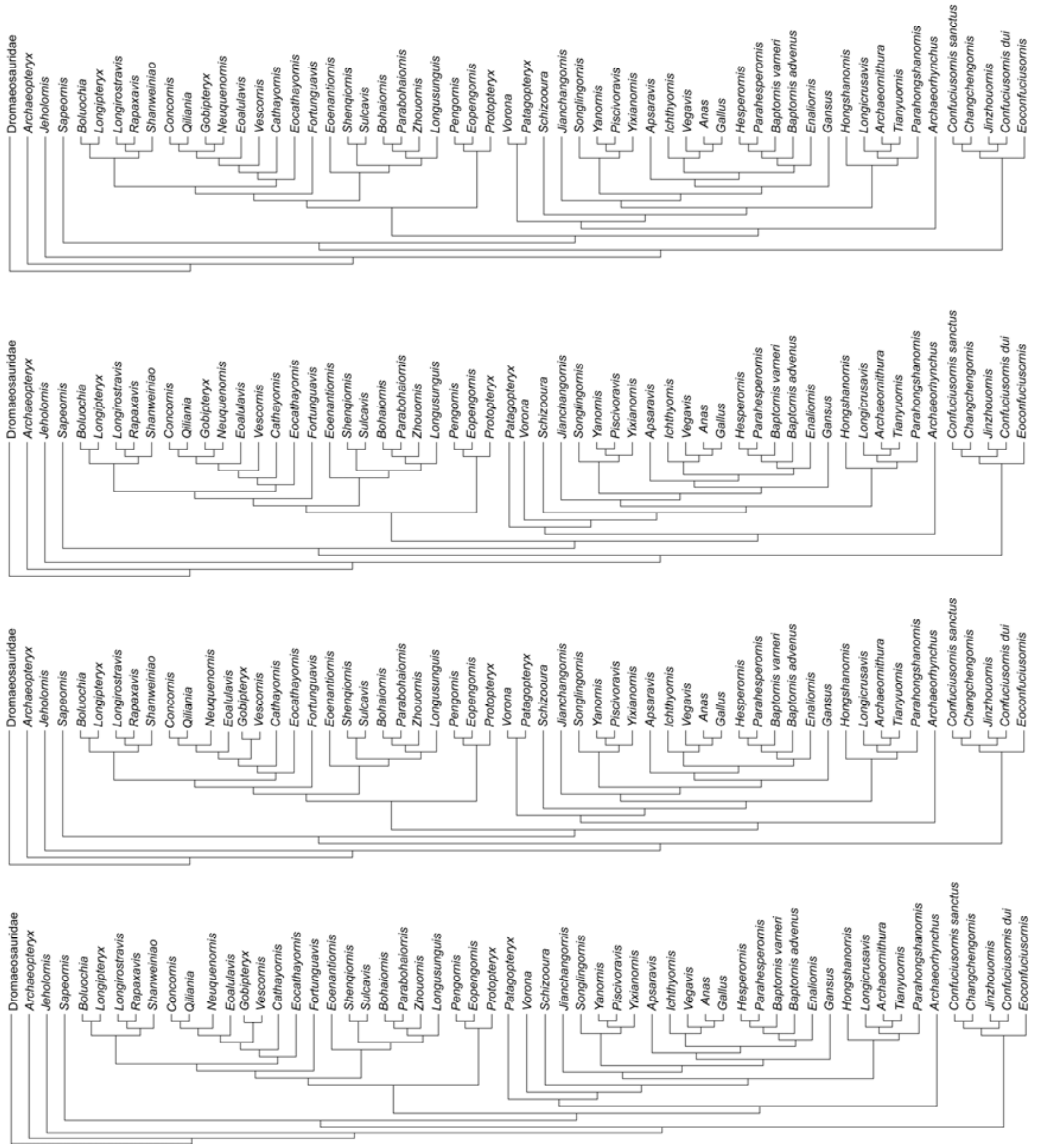

Supplementary Figure 3. The four most parsimonious trees (tree length = 997, CI = 0.367, RI = 0.684) produced from the phylogenetic analysis of the data set of Mesozoic Aves (262 morphological characters, 58 taxa).
