# Supplementary material for: The oldest record of ornithuromorpha from the early cretaceous of China
Source: Nat Commun. 2015 May 5;6:6987. doi: 10.1038/ncomms7987 (PMC5426517; doi:10.1038/ncomms7987)
Supplement: Supplementary Data 2 — Morphological character scorning in the phylogenetic analysis [file ncomms7987-s3.doc]

**Supplementary Data 2**

Character Matrix of 262 Morphological Characters for the 58 Taxa Included in the Phylogenetic Analysis.

Dromaeosauridae 0000000000000000000000000000000000000??00000000000000000000000000000000001????1?00?0000000000?000000000000020?[01]??00100000000000100000000000000100000000??000?000000000000000[01]0000000[01]00000000000010000[01]00?000000000100000000000000000000[01]000000000000?0000000000[01]00000

*Archaeopteryx*  00000000000000?0010010?00?000??0?000???0000000?1000?100000??00000?00020001????0?00?0000000000?0000?0?0000000????????????000?0001100000000000???00000000??001000001000000000000002000000001?0001?010002?10?00000?0001?0??0??00000000000000?0?00?000100?0000000?00100010

*Jeholornis*  [01]?23111????????????0?????????????00?????000?00?1??0????00??10000????010000??????01001?000001??1201?0??1??00[12]?????????????0000000000?0[01]00??01011110?0000012110010000100020000100130000000011?101?010001????0???0?0??0[01]0?10??1100000000??00?0200?0010?00?1?00100101000?0

*Sapeornis*  1010010[01]?0??????0010?1?0?????????1?0????00??0??1????100[01]01??10010?0024000000?00?00?011000000??020?0???10002??????????????00??0001100000???0100111????00??21100001000?0020010101030000??0?110101111?001010??0100?1000?0?111?11000000000000?0000?0011???00?0000000101010

*Confuciusornis sanctus*

10231111?0?????00010110000120???11?1121010?1100?101?101[01]01112001000024000?00001?01?0?0?0000???020?0?00000002[12]10??011210000001000210?000001010000100210001210?000?100?00000002100200000000?101011110003101120101?1?0010000?111000010001?21000000001100?00?0000000000110

*Changchengornis* 1?231111?0????????1??????0???????1?11??0?0??100???[12]?????????2??1???024??0?0000??01?0?0?000????020?0??0?000021?0??01??10?0?0????02??????????????01?????0????0?0000?0??000000021002???0???????????[12]???03???????????0?????????110000?0?0??2??0100?0011?0?00?0?00?00100110

*Eoconfuciusornis* [12]?231?1[01]?0??????0????????????????1?01??000??100????0?0?00???0??1???0241???0??0??00?0???000000?020????0000?02[12]???????????0?00000?100?0???000????01?????0??01??00001?0?0000000210020??0??0?????????1000[12]??????????0??????10????0?000000002?00200?001000???0??00010100100

*Jingzhouornis*  0023111[12]?1??????00???1???????????1?11??010??10???????0??0?1110??????240[12]0[12]?000??0??0???????????00?????000?02[12]10??0?1[012]10?00000??021020[01]0???11?0101??[01]???0?01????01?00?000000021002???????????????????0??012??10??100??0010??110000[01]00??0?1?0200?0?10??????0??001000011?

*Confuciusornis dui*

??23111????????????????0?????????1???????0??1??????????????????[012]????14?10?0??0??0??????????????????????????2[12]?0??0???10?0?0??00021?????????????01?????0???1??0[01]00?00?000??0021002?????????????????????????[02]?????????1???????10????0?00????0[12]00?0010?0????0??0?1000011?

*Boluochia*

?1[12][02]????????????????????????????????????????????????????????????????2????????1?????????????????????????????[12][12]?[12]??????20????????????????????????????????????????????????????????????????????110101???02??????????0???????????0[01]?100100002??3200?0011??1????????2?20????

*Concornis*

????????????????????????????????????????????????????????01??11????????0?01???????111110?02?11?00?1?????1112221222111?20?00111??0[01]0211??????1????1112????????????0??0???30010[01]0122??????????11?1?[12]??00?0?????????1111???????1110?0010??0[01]??0000?001???01131110?0?2??1??

*Eoalulavis*

??????????????????????????????????????????????????00111200??2?0???????????????0??11111010211100000010?111?312?0?0010?40?00111100102110010011111111?21?1?????????0??1?01?001????2???0?????1??????????????1?1????????????????????????????????????????1??1121111?????????

*Cathayornis* [12]0[12]0??0??????????????????????????000????00?????1??1??1??0???11?2????240?0?0111???1111?0??20???000001?0111?322122311??20?0011?1?010211[01]01011111011????110?31100010?[01]00013001010122000000??11??????100????12????1????[01]?????1?1?????????????????????????0?1111111????????

*Eocathayornis*

???0???2???????????????0???0?????000????00?????1????????00???????????????????????11?????02????000000??0????22121311??20??01??1?000???[01]????11???10??????0?31??0010??0001200100011?????????????????????????????????????????????????????????????????????0?111111?????????

*Eoenantiornis* [01]010??02???????????????0?????????00????????????1???????????????[012]????24020?????1?0111110101?11000?001???11122[123]?2??110?2000?1??1?000?????????????11?????10?31??0000?000012001010122???0???????????[12]??001????1?????????????????11??0?10??????0??1?0?1110011[23]1011?[01]?1?0??2

*Gobipteryx* 10[12]3101200021?10????????0???0?1??1?[01]0???10???10??????1?????????[012]10??[12]401?????????1111?010?1???0??0010??111[23]?????????????0??????0??????0????????11???111?????????0??0?01?00??????20???????????????10?03???????????10[01]10?011?110010?100??11?0??1?1??????????2??1????????

*Longipteryx*

?122111??????????????????????????000????00??01?1??1011?????????2????24??0?01?11?01?1110100????000?000?111132[23]?21111??20??0110??0002??[01]?0?011?1?11??????0?01100000100?0120010001130000????1111011[12]11001?0?[12]1?10????0????0?1?1?0?1000000001?32000001???1?1211?1?2?2????0

*Longirostravis* [01]022??1??????????????????????????00?????00??01?1??1?????0?????01????24??0?01110?0111????00?2??000000??0111[123]2[123]?224110?20??01??1??00???[01]?????????10??????0?31??10?0?[01]01012001??0122??0?????1??10?1?11????0?1??????1?00?????1?11[01]?10010??0?2?0100?00????0?1??111?1?1????0

*Neuquenornis*

????????????????????????????????????????????????????????0???11???????????????????1?1????021111000????1?1111230[12]?1?1?????0????11?????1?01?01????11?1??11??31??10?0?[01]0001?????????????????????????????????1110???????1????????10??0?1??1?2?00?011001[12]????1??2[12]0???1?0???

*Pengornis*  00[01]01101?0??????0??????0?1???????000???0?0?????11?2??1??0?1?10?1????240?0[12]???0??01?11???00?????0??02???11?2?????????????001?0100101??[01]?100?1???11??????1[02][23]111???0?01001?0010?0??3???????????????????02?01???????1??[01]?01[12]11?0100100100101200201?00?????011?101?[01]011?021

*Eopengornis* 0000110[012]?0?????????????0?????????00000?000?????1??10?0??00??????????24??0?1?????01211101000???000?02?00111321?110?1??10?0010010000??10?0001????10??????0??1[01]?00?0?010012001010103??????????????????000???????0????100??????0???0001????1??0101?0012?000111101?1011102?

*Protopteryx*  [12]?[12][02]1??[012]?????????????????????????00??????00????1???????10?????01????14????0??1???1111?0?01?[12]??200?00??0111322111011?[12]10???1????010?????????????11?????????1??000??[01]0001100000010???00????11?????????0[12]????1?????0???????????00??0010??00??0[01]01?001110011?0100?[01]101121?

*Rapaxavis* [01]022??1[012]?????????????????????????000???0?0???1?1?????1??01??11?1???024??0?0?110?01????0?00?[12]??0??001???11?322122411??20??0100??0000??[01]0???1111?10???1??0??11?00?010000130011?01230000?0??111101?111?0100????????0?0[01]?0?11??100?10000??002?0100?0011??0?121011?10110210

*Shanweiniao*  [01]?[12][23]??1????????????????????????????0????00??01??????1???????????????24?????1?1??01?11???000[12]??000000??011?22[123]?211?[01]??20???1?????00????????11???11????1?????????????????20?11???22?????????1??????1????0?????[01]0??1??????????110??0000????1?[01][01]?0?0?1[01]?10?12?111?1011?210

*Vescornis*

?0?????2?????????????????????????0000???00[12]001?1??21?1??0???1??2????2???????00??01?1??0?02?[12]??000001??011?322120111??20?00110??0101?1[01]0???1111111??????1[02]011010?00[01]0?0130010[01]01220??0???????????????????????????0??[01]????????00??0010??011?[01]201?1011??11121[01]11?[01]1110?00

*Vorona*

??????????????????????????????????????????????????????????????????????????????????????????????????????????????????????????????????????????????????????????????????????????????????????????????????????101120111?0001111011111[12]000100011?21000000?????????????11???????

*Schizooura*  0123111[01]????????????????????????11?0????10??01??0?[12]1?00?00?1??0[345]?1??[12][34]?[12]0[12]?000??01011?0?1001??[01]2?00????11012[23]0[12]?011??1??1?000??0[12]0010[01]0???01?0?10?????00?31??0000?001?0[23]001010103?0??1[01]1???0101?1?0103?????[12]?0?????[12]???????11?010?00001[01]2?011000010?1?01?1110?10000001

*Jianchangornis*

???????????????????????????????????????1??????????1?????010?200[234]0???????????????0101110010020?0200?0?0121?02[23]031001??[01]??01000000100100000001?0111?????0???1??01?00000002101010103??01?[01]?????????[12]?000????????00???0??0?10??1120?00?0??????0100?00???01010001110000?0?1

*Archaeorhynchus*  1013111[12]?????????????????????????1?00??00?00??1???0?????0??1???2000??[34]0111??????01011?00?0?20?[01]20000?0?21002[23]122211?[12]5???1000??010????0???0110?10????101?01??000??0??0?21010???230000????101100?[12]10001??????????0?0[12]00????211[01]010000????0?0100?00????0010101100000?01[01]

*Songlingornis*

???[01]?????????????????????????????000?????0?????1????????????2????????????????????10?????101???2????0???21?02301231[12]??51?????????????????????????????????????????????????????????????????????????????0????????????????????????????????????????????????1?10?01??????????

*Apsaravis*

??????????????????????????1[01]?????1?1????1????????021?1?201??00?4??0?24?10????????1011100001111121102001????[12]30???11?????11001000101110000111111110121101?311001110110???1????????100011111?111012??113101[12]?110??1???20121??11[12]?111001?1?200100?00??????1??12110??0????

*Yanornis* 10[12]11102?1????????1?????????????1000???1?00?01?1??0?????01??2003??0?????????????01011110100?0?220001??121?02301121[12]??51011001??010110[01]????0100011?0?11?0?31110000000010210101012310100?11?10100?11?003??1???????1?0??0??0??111011?00[01]??12?0100?0011??0?101001000000000

*Patagopteryx*

????????????????1?11110001111????????????0??000?11211011001100031101??120????????1011100000?0?1210010??????2[12]????10?????1001???1000?0?00000100?0000?0?0?????????0??1?1?20?00???20?0001000001110121?013?0??20????1001?0?01111120101001110000?00?0001????10??110100000?0

*Yixianornis* 1121???????????????????0???1?1???0?0???110?????1??1?1??2011?1003??0?24????1000[12]00101110010010?220001001210?2301121[12]0?51??10??11??0?0?0?11001?0?100021?0??3111010100001?2101010102100?1??00?01??11??003??1?2?101?100????????11[12]01?0001110?0010??0011?110101011?0000001?

*Gansus*

?????????????????????????????????????????????????121?1?201111004001024?20210000?1101110010020?320???0012100230210100?10?11001??010130?1???010011100111?0?312?01110001?131010001321?0011110101011210003001[12]?1101?100[12]2?0?0?211201110010102?020000011??001?10110?010000?

*Ichthyornis* 21[12]???0?????????111111??1111???01001???10021011111[01]11012011120?[34]?0102401021000???101111010010?2210110012?002300??1212?011100110010120210?101000110021101?3121012101001031111????310?01011??1101121?11?101221101?100221110?2112011202?01?210200000??????10101001?1????0

*Hesperornis* 11231103?1021011?111111000111?1?10100??100010101?021001211112004010?130202100010?0?01?00?0010?020???000200?21?0??0011?00??????0?????1000000??????????????????????????????????????1000121101110012101131021222011101221111121120112?21013223220102??????0??01?020?0?0?0

*Parahesperornis* 1123??0???0?10??1?111???00111????01????1???????1?02100121111200?????1?????10001??0?01?0??0010?0????????????2???????1??????????????????????????????????????????????????????????????????????????????????1021222011101221111???1201?2?2101?223220002?????????????20?0?0??

*Enaliornis*

??????????????????????????11??????????????????????21?0??1???2????????????????????????????????????????????????????????????????????????????????????????????????????????????????????1??????1???????2?????1012212011101221111???1201???1101???0120000????????????02???????

*Baptornis advenus*

1??3??????????????11???00?111???????0??1?0?10101?021001?11112004010?13020?100011?0001?0010010?0?????0??????2[12]????0112??0????????????1000000????00000100?????????????????????????010001211011100121011310112120111012211111211201?2?11013221120001????????????02???????

*Baptornis varneri*

?????????????????????????????????????????????????021?01?1??1?????????????????????????????????????????????????????????????????????????????????????????????????????????????????????10????1???1100?210113?????2201?1012211111211201?2?1101?221220001?????????????2???????

*Vegavis*

??????????????????????????????????????????????????2????????????[56]?????????????????1011?00?001??[01]20?00????????????????????1?0???????????????????????????0??????????????????????????01???1?????01??2??????0112110??1?02?2?1????12011???3?1????1???0???????10?01??????????

*Anas*  2123011310111111111111111112112111?102100001011?102110121111000601202402021000201101111010020?220100001210023111001134011100111100030111110100001001110123120132111111031011??1231101111101011112101131011211011100222011121120112013013210220000101110101121010100010

*Gallus*  1123111311121111111111111112112111?102000001011?102110121111001601202402021000201121111010020?221100001210123110101122011100111100030111111100011001110113120132102101131011??1211101121101101112101131011211011100222011121120112013013220120100101110101121010100010

*Fortunguavis*

??????0?????????????????????????????????00???0????0?110200??1?000??02[34]??0??1110?01111101001110020????0111122??2??1???300000??10000??1??0001?1??11?????112211000001001012001000?120?????????01011110012?0????????1???10???1?110010010000?0?02?100011???11?10?1?11100111

*Qiliania*

?????????????????????????????????????????????????????????????????????????????????????????????????????????????????????????????????????????????????????????????????????????????????10????00?10101?2?0003?0??11100?00?110000?11100?001001?11?[01]?20?0?1[01]??????????1?120021?

*Shenqiornis* [01]0101102????????0??????0?1???????000???000???1?1???11?0?01??11??????????0???????01?11?0?01?[12]??010101??011?[23]2[123]?[12][12]3?1??20??01101?000?????????1???11????????01??001010010120010101130000??????01?111???01??????????0????????????0?1011???0???[01]?0?0?1???[12]111211[12]01??2?0?21

*Sulcavis* [01]010??0????????????????0?????????000???0?0?????1?????11?????11?[12]????24?1010110??01????0?010[12]??0[01]0001?0?1113[12][123]????????????01????010?????????1???11??[01]???0?[02]1??0[01][01]0?00?0120010101120?00?[01]?????????[12]10?????????????0?00?0?01??110010010??0[123]??0101?0011???11211[12]011110012?

*Bohaiornis*

?0[12]01102?0?????????????0????????1000???000??0011????1?120?1?110[0123]????24000?01101?0111110?01020?0[01]0101?0?11122[123]?21311??30?001??100001?10?0?011???11?????10?0110000010010120010101230000??????01011[12]??000?0?1????0?0??0?0??10?1100100100?01?[01]0101?0?11??10121021111000121

*Parabohaiornis* [01]0201102?0???????????????????????000???000??0??1???01??200??110[12]01?124??0?01101101?1110?01?11?010101?0111132[123]?20311?[345]30?001??10000101010001110111?????10?01??000010010120010101230000??0?0101011[12]1100000??????0?0?00?00010?100010010??01200101?0?11??1112102111100022?

*Longusunguis*

?0[12]01?02?0???????????????????????000???000??01?1??[12]?1??20??1110[123]????24?00?0110??01111101020???010001?0111?32[123]????1???[23]00001??10000??10?00011???11?????10?01??0000100101?00101012200????????01011[12]??000????1?100?0?00?00010?100?1???000?1??010100?1????112102011?100221

*Zhouornis*  [01]0101?01?0???????????????????0??1000???000??0??11?[12]?1??20???110?????24??0??1?0??011111010101??020101?0111132[123]?2?3110??0?000??10000??10?00011???11?????10?31??000010010120010101220000???????????[12]??00000??1?1?0?1?00?0?01??110010010??01??0101?0?11??11121?2011110012?

*Piscivoravis*

???????????????????1????111?[01]???????????0?????0??12110110111210[34]????24020??0001101?11?0??0????[23]20????0121002301221???51?0?0????1????0??????1?0?11?????00?31??00011000102101010112101010????01011210003?01121?01?10?22????1?112011100[01]1?02001100001011001?01???0001000?

*Hongshanornis*

?020?10??????????1????????????????00???101??0???????????????1???????24???????01?0101110010????[01]211?0?0121?12??2201[12]?210?110?????10???2????0????11????????31??00?0??00?02100010101??????????0??01[12]????3???????????0?[12]???????1120?0?00?0?0??0200?00111100101011001100001

*Longicrusavis*  10[12]01?0???????????????????????????00???101??01????11?11?0???20???????????????????1011100??????[12]21100?01210?23?120110?10?110?01?11012021100010001100???0??31110??00010102100010101???????11??1???[12]??0?????????01?10022[01]121?1112011100??10100100?0011??0010101100110000?

*Archaeornithura*

???????????????????????????????????????????????????????????????[23]???0[12]4??1110001?0101110010110?221100?0?2101230[12]?111???????0??10010??02????0100????????0??[23]1??0[12]?0?000?02100010101?0????????01001[12]???03????2??0??1?0????2?0?1110?1?0????[01]??0100?001111?0101011?01100001

*Parahongshanornis*

????????????????????????????????????????????????1?1??????????????????????????????1????0?10????[01]211???01210123022011??10??10??1?010??0?????01???11????????31??01?0?000102100010101?0?????????10??[12]??003???????0????00???????1???11?0????1??0100?0011??001?1011?0110000?

*Tianyuornis* 1020??0??????????????????????????000????01??01?1?????????????????????4????????11?10???0?10????22??????1210123?22111??10?1100???010??0?????01?0?11????????[23]1??0??0?000102100010101???????????????????????????????1??[12]??12???111011?0????[01]??01?0?0?11??001?1011?0110000?
